# Biological age acceleration and cardiovascular–kidney–metabolic syndrome progression: evidence from UK Biobank and NHANES

**DOI:** 10.1080/0886022X.2026.2738248

**Published:** 2026-10-11

**Authors:** Yukai Zhang, Chenwei Zhang, Xin Wang, Yun Zhou

**Affiliations:** a Department of Nephrology, The First Hospital of Shanxi Medical University, Taiyuan, Shanxi, China; b Department of Health Statistics, School of Public Health, Shanxi Medical University, Taiyuan, China

**Keywords:** Cardiovascular–kidney–metabolic syndrome, PhenoAge, Klemera–Doubal method biological age, biological aging

## Abstract

**Objective:**

We examined associations of biological age acceleration with cardiovascular–kidney–metabolic (CKM) syndrome progression and baseline stage burden.

**Methods:**

Longitudinal analyses included 326,218 UK Biobank participants, and cross-sectional analyses included 5,228 NHANES adults. PhenoAge and KDMAge acceleration were residuals from regressing each biological age measure on chronological age. We modeled two UK Biobank transitions using cause-specific Cox and competing-risk models and compared NHANES stages using survey-weighted logistic regression.

**Results:**

Over a median 13.48 years, 2,567 participants progressed from stage 0/1 to stage 2/3 and 42,835 from stage 1 or 2/3 to stage 4. Per 1-SD increment, PhenoAge acceleration was associated with both transitions [hazard ratios (HRs) 1.234, 95% confidence interval (CI) 1.189–1.281; HR 1.208, 95% CI 1.197–1.218]. KDMAge acceleration showed corresponding HRs of 1.229 (95% CI 1.180–1.280) and 1.220 (95% CI 1.207–1.232), all *P* < 0.001. Results remained directionally consistent after accounting for competing mortality. Associations for dichotomized accelerated aging remained significant after false discovery rate correction, and UK Biobank splines suggested possible non-linearity. In NHANES, PhenoAge acceleration was associated with stages 1, 2/3, and 4 versus stage 0. Corresponding odds ratios (ORs) were 1.154 (95% CI 1.107–1.202), 1.221 (95% CI 1.176–1.268) and 1.333 (95% CI 1.249–1.423), all *P* < 0.001. KDMAge acceleration ORs were 1.025 (95% CI 1.006–1.044; *P* = 0.012), 1.038 (95% CI 1.019–1.057; *P* < 0.001), and 1.041 (95% CI 1.011–1.073; *P* = 0.010).

**Conclusions:**

Biological age acceleration was associated with CKM progression and baseline burden. KDMAge comparability remains uncertain. Incremental predictive and clinical utility remained untested.

## KEY MESSAGES

**What is known:**

Biological age acceleration is associated with cardiometabolic and renal outcomes, but its relationship with CKM stage progression remains insufficiently characterized.

**What this study adds:**

PhenoAge and KDMAge acceleration were associated with CKM progression in the UK Biobank and greater baseline CKM stage burden in NHANES.

**Potential impact:**

Biological age acceleration may help characterize CKM burden and progression risk and warrants further evaluation as a potential complementary marker.

Clinical trial number: Not applicable.

## GRAPHICAL ABSTRACT

**Figure:**
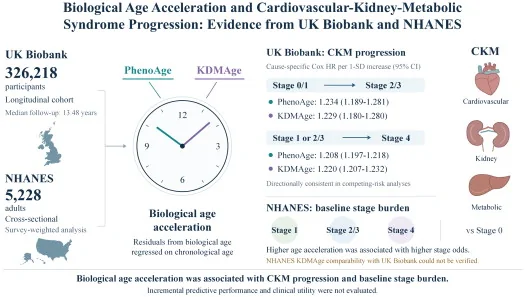

## Introduction

Cardiovascular–kidney–metabolic (CKM) syndrome describes the interconnected burden of adiposity, diabetes, chronic kidney disease (CKD) and cardiovascular disease (CVD), which collectively contribute to multiorgan dysfunction and adverse cardiovascular outcomes [1,2]. The CKM framework conceptualizes this burden as a clinical continuum extending from the absence of risk factors to established clinical CVD, highlighting the progressive convergence of metabolic, renal, and cardiovascular disorders [2]. Advancing CKM stage carries important prognostic implications, as higher stage burden and the coexistence of diabetes and kidney disease are associated with increased risks of cardiovascular events and mortality [3,4]. In the U.S. population, diabetes and CKD were each associated with elevated 10-year mortality, whereas their coexistence was linked to substantially greater 10-year mortality risk [4]. Therefore, examining factors associated with CKM stage progression may improve understanding of this disease continuum [3].

Population aging is accelerating worldwide, with projections indicating that the number of adults aged 65 years or older will exceed 1.6 billion by 2050 [5]. Aging is a major driver of cardiometabolic, renal, and CVD burden, with metabolic syndrome being particularly prevalent in older populations [5,6]. Progressive CKD shares several biological hallmarks with renal aging, including cellular senescence and impaired tissue repair [7]. Biological age was developed to capture inter-individual heterogeneity in physiological aging beyond chronological age and has been associated with morbidity and mortality across adult populations [8,9]. Among existing biological age measures, PhenoAge and KDMAge are blood chemistry-based algorithms that integrate routinely measured biomarkers and can therefore be applied in large population-based cohorts [9,10].

Biological age acceleration denotes a biological age that exceeds that expected for chronological age and has been associated with age-related disease burden and mortality [11]. Although PhenoAge and KDMAge have been linked to cardiometabolic multimorbidity, renal outcomes, and mortality, their specific relationships with CKM stage burden and longitudinal CKM progression remain insufficiently characterized [12,13]. Accordingly, we used the UK Biobank as the primary longitudinal cohort to evaluate whether baseline PhenoAge and KDMAge acceleration were associated with subsequent CKM progression. NHANES was further used as an independent population-based cross-sectional cohort to examine associations between biological aging and baseline CKM stage burden in a U.S. population sample.

## Methods

### Study design and population

This study used a two-cohort design, with the UK Biobank as the primary longitudinal cohort and NHANES as an independent population-based cross-sectional cohort. The UK Biobank is a population-based prospective cohort that enrolled approximately 500,000 participants aged 40–70 years from 22 assessment centers across England, Scotland, and Wales. Baseline assessments were conducted between 2006 and 2010 and included questionnaire data, physical measurements, laboratory testing, and linkage to health-related records. The present analysis was conducted under UK Biobank application number 778815.

In the UK Biobank, we examined whether biological aging at baseline was associated with subsequent CKM progression. Follow-up accrued from the baseline assessment date until the first documented CKM progression event, death, loss to follow-up, or the country-specific administrative censoring date, whichever occurred first. Administrative censoring dates were defined as 31 October 2022 for England, 31 July 2021 for Scotland and 28 February 2018 for Wales. Participants were eligible for the longitudinal analytic sample if they had complete data on PhenoAge, Klemera–Doubal method biological age, baseline CKM stage, and country-specific follow-up information.

NHANES is a cross-sectional surveillance program administered by the U.S. Centers for Disease Control and Prevention. It uses a stratified, multistage probability sampling design across biennial cycles to characterize the health and nutritional status of the U.S. civilian, non-institutionalized population. In this study, the three nonconsecutive cycles represented in the available biological age dataset (2005–2006, 2007–2008, and 2015–2016) were used to examine the cross-sectional association between biological aging and baseline CKM stage burden in an independent population. Participants were included in the survey-weighted NHANES analysis if they had complete demographic data, variables required for biological age estimation and CKM staging, model covariates and a positive fasting subsample weight. Owing to its cross-sectional design, NHANES was used only to assess baseline associations and not CKM progression.

### Calculation of biological senescence and its acceleration

Biological aging was quantified using two validated blood chemistry-based indices, phenotypic age (PhenoAge) and Klemera–Doubal method biological age (KDMAge), according to previously published algorithms. PhenoAge was calculated from chronological age and nine routinely measured biomarkers: albumin, alkaline phosphatase, creatinine, C-reactive protein, glucose, mean corpuscular volume, red cell distribution width, white blood cell count, and lymphocyte percentage [10]. KDMAge was estimated using the Klemera–Doubal method, which incorporates physiological and biochemical markers related to pulmonary function, blood pressure regulation, metabolic status, renal function, and systemic inflammation. Variables used for KDMAge estimation included forced expiratory volume in 1 s, systolic blood pressure, albumin, alkaline phosphatase, blood urea nitrogen, creatinine, C-reactive protein, glycated hemoglobin, and total cholesterol. Biomarker transformations and unit harmonization followed the original algorithms, with further details provided in Supplementary Method 1. Creatinine, glucose, blood pressure, glycated hemoglobin, and cholesterol overlap with CKM staging; the validated algorithms were retained intact.

PhenoAge acceleration and KDMAge acceleration were derived as residuals from cohort-specific linear regression models in which each biological age measure was regressed on chronological age. Positive residuals indicated biological age greater than expected for a given chronological age and were classified as accelerated biological aging. Residuals equal to or below zero were categorized as non-accelerated aging. These age acceleration measures were analyzed as continuous variables and, where appropriate, as categorical indicators.

### Definition of CKM syndrome

CKM syndrome was defined according to the framework proposed by the American Heart Association. Briefly, stage 0 denoted the absence of CKM risk factors; stage 1 reflected excess or dysfunctional adiposity without additional metabolic risk factors or CKD; stage 2 was defined by the presence of metabolic risk factors or CKD; stage 3 corresponded to subclinical CVD or high predicted cardiovascular risk; and stage 4 indicated established clinical CVD. Detailed operational definitions are provided in Supplementary Method 2.

In the present analysis, stages 2 and 3 were combined into a single intermediate CKM category. This approach was adopted because direct and harmonized information required to define subclinical CVD, the defining feature of stage 3, was not consistently available across both cohorts. The use of cohort-specific surrogate definitions could have introduced additional misclassification and reduced comparability between the UK Biobank and NHANES.

### Outcome ascertainment in the UK Biobank

CKM progression in the UK Biobank was ascertained using linked health-related records. Progression from stage 0 or 1 to the combined stage 2/3 category was defined as the first incident diagnosis of diabetes, hypertension, or CKD during follow-up. Progression from stage 1 or the combined stage 2/3 category to stage 4 was defined as the first incident clinical CVD event. Clinical CVD included coronary heart disease, heart failure, stroke, peripheral artery disease, and atrial fibrillation. For each transition, the earliest qualifying diagnosis or event date was assigned as the event date. In cause-specific Cox analyses, participants without the transition of interest were censored at death, loss to follow-up, or the country-specific administrative censoring date, whichever occurred first. In complementary competing-risk analyses, death preceding the corresponding CKM progression event was treated as a competing event.

### Selection of covariates

Covariates were prespecified based on prior evidence and their availability within each cohort. In the UK Biobank, primary longitudinal models were adjusted for baseline age, sex, ethnicity, socioeconomic status, educational attainment, smoking status, alcohol consumption, and physical activity. Socioeconomic status was represented by the Townsend Deprivation Index, an area-level measure derived from unemployment, household overcrowding, car ownership, and housing tenure. Physical activity was quantified using total metabolic equivalent task scores.

In the NHANES analysis, cross-sectional models were adjusted for age, sex, race, and ethnicity, educational attainment, marital status, socioeconomic status, smoking status, and alcohol use. Socioeconomic status in NHANES was captured by the poverty income ratio, defined as household income relative to the federal poverty threshold.

Variables directly contributing to CKM stage definitions, including body mass index, hypertension, diabetes, CKD, and CVD, were not included as covariates in the primary models to reduce overadjustment. Where applicable, medication use was incorporated into the operational definitions of CKM-related conditions. Medication variables were therefore not additionally adjusted for in the primary models, as further adjustment could attenuate the estimated associations through overadjustment.

### Missing data

Participants with missing biomarkers required for PhenoAge or KDMAge estimation were excluded from analyses of biological aging. In the UK Biobank, missing continuous covariates were imputed using sex-specific means, whereas missing categorical covariates were coded as an unknown category. Complete-case analyses for covariates were conducted as sensitivity analyses. In NHANES, the survey-weighted primary analysis was restricted to participants with complete data on biological aging measures, CKM staging and covariates and a positive fasting subsample weight. To evaluate potential missing-data mechanisms in NHANES, analytic-sample inclusion was defined as a binary outcome among participants with available source data for CKM classification. Inclusion rates were first examined across survey cycles. Among participants from cycles represented in the biological age dataset, survey-weighted logistic regression was then fitted with age, sex, race and ethnicity, survey cycle, and observed histories of hypertension and diabetes as predictors. The model incorporated masked strata, primary sampling units, and pooled examination weights. A second inclusion model was restricted to the 6,549 participants with available biological age model data.

### Statistical analysis

Baseline characteristics were summarized according to CKM stage separately in the UK Biobank and NHANES. UK Biobank continuous variables are presented as means with standard deviations (SDs) or medians with interquartile ranges, and categorical variables as counts and percentages. NHANES summaries incorporated the complex survey design and are presented as weighted means or medians and unweighted counts with weighted percentages. Across-stage comparisons in NHANES used survey-weighted regression tests for continuous variables and design-adjusted chi-square tests for categorical variables.

In the UK Biobank, cause-specific Cox proportional hazards models were fitted to examine the associations of PhenoAge acceleration and KDMAge acceleration with incident CKM progression. Two prespecified transitions were evaluated: progression from stage 0 or 1 to the combined stage 2/3 category, and progression from stage 1 or the combined stage 2/3 category to stage 4. Hazard ratios (HRs) and 95% confidence intervals (CIs) were estimated per 1-SD increment in biological age acceleration. Additional analyses assessed quartiles of biological age acceleration and accelerated aging status, defined as residual values greater than 0 versus values equal to or below 0. The proportional hazards assumption was evaluated using Schoenfeld residuals. Complementary Fine–Gray models, adjusted for Model 3 covariates, treated death before CKM progression as a competing event. Each model used the full transition-specific risk set and participant-clustered robust variance to estimate sHRs and 95% CIs per 1-SD increase in biological age acceleration.

In NHANES, survey-weighted stage-specific logistic regression models compared CKM stage 1, stage 2/3, and stage 4 with stage 0. Because fasting glucose and triglycerides were components of CKM staging, analyses applied the pooled fasting subsample weight (WTSAF2YR/3) and accounted for SDMVSTRA and SDMVPSU using Taylor linearization. Absolute biological age and biological age acceleration were modeled separately, with acceleration estimates presented in the main text. Given identical analytic samples, covariates, and linear adjustment for chronological age, the two parameterizations yielded algebraically equivalent exposure coefficients. Results are expressed as survey-weighted odds ratios (ORs) with 95% CIs and were interpreted as cross-sectional baseline associations rather than progression. To examine this discordance, we fitted *post hoc* diagnostic models without covariate adjustment and with sequential adjustment for age and sex, and Model 3 covariates. We also assessed quadratic and spline age specifications and summarized survey-weighted mean KDMAge acceleration by sex and female proportions across CKM stages.

Three sequential adjustment models were specified in both cohorts. In the UK Biobank, Model 1 adjusted for age and sex; Model 2 further adjusted for ethnicity and the Townsend Deprivation Index; and Model 3 additionally included educational attainment, smoking status, alcohol consumption, and physical activity. In NHANES, Model 1 adjusted for age and sex; Model 2 further adjusted for race and ethnicity and the poverty income ratio; and Model 3 additionally included educational attainment, marital status, smoking status, alcohol use, and survey cycle.

Restricted cubic spline models with knots placed at the 25th, 50th, and 75th percentiles were used to evaluate potential non-linear associations between biological age acceleration and CKM progression in the UK Biobank. Sensitivity to knot placement was assessed by repeating the fully adjusted UK Biobank Cox spline models with three or four knots. Likelihood-ratio tests evaluated overall and non-linear associations using the same standardized exposures, outcomes, and Model 3 covariates as the primary models. UK Biobank sensitivity analyses excluded participants who developed CKM progression within the first year of follow-up and repeated the main analyses among participants with complete covariate data. UK Biobank subgroup analyses were conducted by sex, age group, ethnicity, and baseline CKM stage, where applicable. False discovery rate correction using the Benjamini-Hochberg method was applied to the primary UK Biobank association tests. All analyses were performed using R software versions 4.4.0 and 4.6.1. Two-sided *P* values below 0.05 were considered statistically significant.

## Results

### Study population and data completeness

Among 502,175 baseline participants extracted from the UK Biobank, incomplete biomarker data for PhenoAge and KDMAge estimation were observed in 92,286 (18.4%) and 152,891 (30.4%) participants, respectively (Table S1). For KDMAge, the largest contributors to missingness were forced expiratory volume in 1 s (48,778 participants, 9.7%) and automated systolic blood pressure (30,110 participants, 6.0%). Overall, 334,987 participants had complete biomarker data required for both PhenoAge and KDMAge estimation. After excluding 53 participants with incomplete baseline CKM staging and 8,716 participants without country-specific follow-up information, 326,218 UK Biobank participants were retained for the longitudinal analysis (Figure 1 and Table S2). Compared with participants excluded because of incomplete biological aging inputs, those included in the UK Biobank biological aging analysis had broadly similar baseline characteristics, with only small differences in age, socioeconomic status, body mass index, blood pressure, and follow-up time (Table S3). Missing continuous covariates were imputed using sex-specific means, whereas missing categorical covariates were coded as an unknown category (Tables S4 and S5). Complete-case analyses for covariates were conducted as sensitivity analyses.

**Figure 1.**
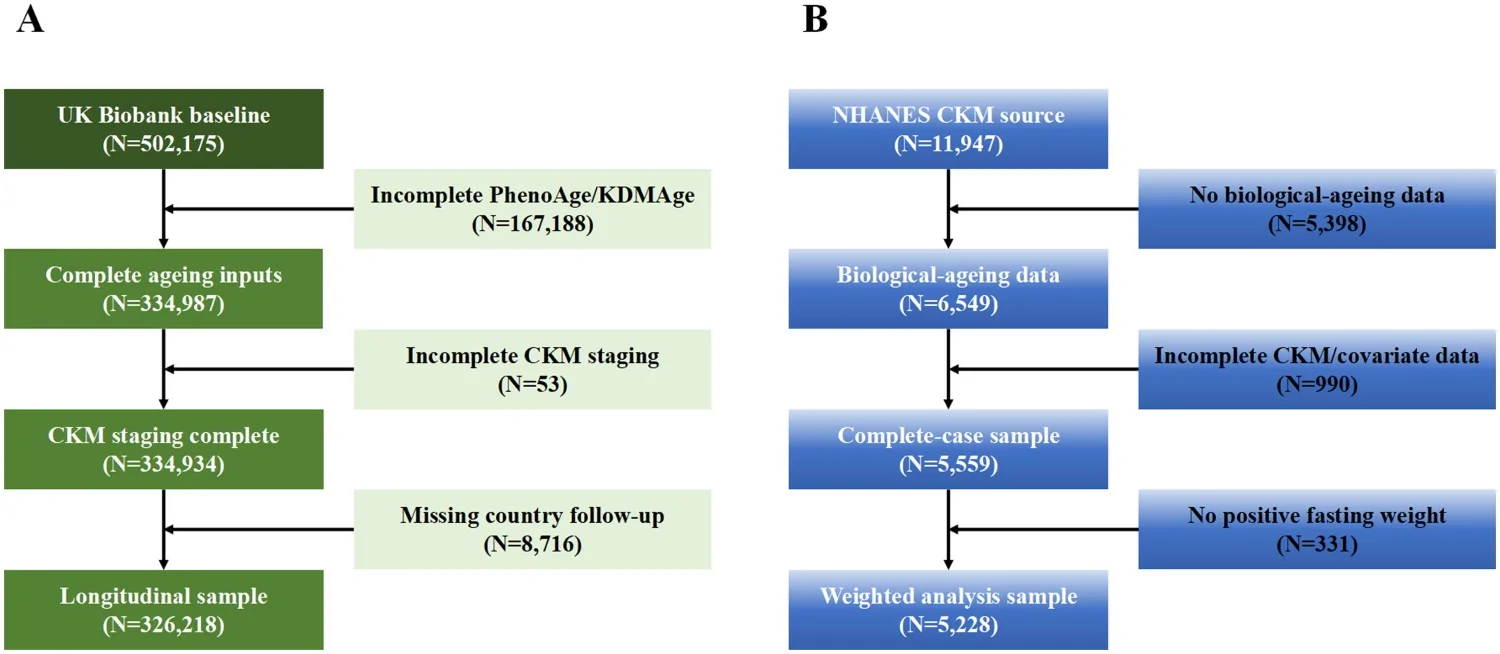
Flowchart of participant selection in the UK Biobank and NHANES cohorts. (A) In the UK Biobank, 502,175 baseline participants were identified from the extracted dataset. After excluding participants without complete PhenoAge or KDMAge measurements, incomplete baseline CKM staging, or missing country-specific follow-up information, 326,218 participants were included in the longitudinal analysis. (B) In NHANES, 11,947 participants with CKM diagnostic source data were identified; 6,549 had biological age model data, 5,559 met the original complete-case criteria, and 5,228 additionally had positive fasting subsample weights for the survey-weighted primary analysis. CKM, cardiovascular–kidney–metabolic; KDMAge, Klemera–Doubal method biological age; NHANES, National Health and Nutrition Examination Survey.

In NHANES, 5,559 adults met the original complete-case criteria; 5,228 additionally had a positive fasting subsample weight and formed the survey-weighted primary analysis sample (Figure 1 and Tables S6 and S7). Before the final complete-case and positive-weight restrictions, missingness in the available NHANES dataset was mainly observed for the poverty income ratio and alcohol use, whereas educational attainment and marital status had lower proportions of missing data. Detailed participant selection is shown in Figure 1.

Analytic-sample inclusion varied by observed data availability and participant characteristics; two source cycles lacking biological age model data contributed no participants to the final sample. Among the 6,549 participants with biological age model data, the survey-weighted joint test of observed covariates was significant (*P* < 0.001); age, race and ethnicity, survey cycle, and hypertension history were associated with inclusion. These findings were inconsistent with missing completely at random and compatible with, but insufficient to establish, missing at random conditional on observed information (Table S8).

### Baseline characteristics

The distribution of baseline CKM stages differed between the two cohorts. In the UK Biobank, 26,874 participants were classified as stage 0, 21,948 as stage 1, 253,494 as the combined stage 2/3 category, and 23,902 as stage 4. In the survey-weighted NHANES analysis, the corresponding unweighted counts were 516, 1,284, 2,923, and 505, respectively. Participants with more advanced CKM stages were generally older and had less favorable clinical profiles. Weighted NHANES and unweighted UK Biobank baseline characteristics are presented in Tables 1 and 2.

**Table 1.** Baseline characteristics of UK Biobank participants according to baseline CKM stage.

Characteristic	Overall (*N* = 326,218)	Stage 0 (*N* = 26,874)	Stage 1 (*N* = 21,948)	Stage 2–3 (*N* = 253,494)	Stage 4 (*N* = 23,902)	*P* value
*Demographics and socioeconomic factors*						
Age, years, mean (SD)	56.43 (8.09)	51.52 (7.63)	52.59 (7.94)	56.82 (7.91)	61.39 (6.38)	<0.001
Sex, *n* (%)						<0.001
Female	176,347 (54.1)	20,900 (77.8)	15,974 (72.8)	131,150 (51.7)	8,323 (34.8)	
Male	149,871 (45.9)	5,974 (22.2)	5,974 (27.2)	122,344 (48.3)	15,579 (65.2)	
Unknown	0 (0.0)	0 (0.0)	0 (0.0)	0 (0.0)	0 (0.0)	
Ethnicity, *n* (%)						<0.001
White	308,552 (94.6)	25,818 (96.1)	20,330 (92.6)	239,769 (94.6)	22,635 (94.7)	
Asian or Chinese	7,251 (2.2)	305 (1.1)	591 (2.7)	5,717 (2.3)	638 (2.7)	
Black	4,315 (1.3)	200 (0.7)	489 (2.2)	3,378 (1.3)	248 (1.0)	
Mixed	1,882 (0.6)	231 (0.9)	194 (0.9)	1,372 (0.5)	85 (0.4)	
Other	2,754 (0.8)	232 (0.9)	249 (1.1)	2,106 (0.8)	167 (0.7)	
Unknown	1,464 (0.4)	88 (0.3)	95 (0.4)	1,152 (0.5)	129 (0.5)	
Townsend deprivation index, mean (SD)	−1.40 (3.01)	−1.49 (2.94)	−1.33 (3.01)	−1.45 (2.98)	−0.88 (3.26)	<0.001
Education, *n* (%)						<0.001
College or university	105,406 (32.3)	12,472 (46.4)	8,164 (37.2)	79,408 (31.3)	5,362 (22.4)	
Other qualification	163,373 (50.1)	12,478 (46.4)	11,385 (51.9)	128,439 (50.7)	11,071 (46.3)	
None of the above	53,742 (16.5)	1,753 (6.5)	2,203 (10.0)	42,732 (16.9)	7,054 (29.5)	
Unknown	3,697 (1.1)	171 (0.6)	196 (0.9)	2,915 (1.1)	415 (1.7)	
Household income, *n* (%)						<0.001
<18,000	61,472 (18.8)	3,169 (11.8)	3,106 (14.2)	47,575 (18.8)	7,622 (31.9)	
18,000–30,999	70,976 (21.8)	4,843 (18.0)	4,289 (19.5)	56,318 (22.2)	5,526 (23.1)	
31,000–51,999	73,658 (22.6)	6,648 (24.7)	5,532 (25.2)	57,645 (22.7)	3,833 (16.0)	
52,000–100,000	57,875 (17.7)	6,754 (25.1)	4,804 (21.9)	44,088 (17.4)	2,229 (9.3)	
>100,000	15,379 (4.7)	2,383 (8.9)	1,494 (6.8)	10,942 (4.3)	560 (2.3)	
Unknown	46,858 (14.4)	3,077 (11.4)	2,723 (12.4)	36,926 (14.6)	4,132 (17.3)	
Employment status, *n* (%)						<0.001
Employed	189,511 (58.1)	20,124 (74.9)	15,866 (72.3)	145,691 (57.5)	7,830 (32.8)	
Retired	107,369 (32.9)	4,016 (14.9)	3,980 (18.1)	86,417 (34.1)	12,956 (54.2)	
Unemployed	5,730 (1.8)	404 (1.5)	374 (1.7)	4,530 (1.8)	422 (1.8)	
Unable to work	9,337 (2.9)	396 (1.5)	421 (1.9)	6,474 (2.6)	2,046 (8.6)	
Other	12,677 (3.9)	1,823 (6.8)	1,186 (5.4)	9,157 (3.6)	511 (2.1)	
Unknown	1,594 (0.5)	111 (0.4)	121 (0.6)	1,225 (0.5)	137 (0.6)	
*Lifestyle and anthropometry*						
Smoking status, *n* (%)						<0.001
Never	179,263 (55.0)	16,839 (62.7)	12,766 (58.2)	139,898 (55.2)	9,760 (40.8)	
Previous	112,356 (34.4)	7,488 (27.9)	6,842 (31.2)	86,884 (34.3)	11,142 (46.6)	
Current	33,060 (10.1)	2,470 (9.2)	2,248 (10.2)	25,518 (10.1)	2,824 (11.8)	
Unknown	1,539 (0.5)	77 (0.3)	92 (0.4)	1,194 (0.5)	176 (0.7)	
Alcohol frequency, *n* (%)						<0.001
Never	25,130 (7.7)	1,679 (6.2)	1,624 (7.4)	18,990 (7.5)	2,837 (11.9)	
Special occasions only	36,845 (11.3)	2,603 (9.7)	2,716 (12.4)	28,354 (11.2)	3,172 (13.3)	
1–3 times/month	36,190 (11.1)	3,155 (11.7)	2,950 (13.4)	27,602 (10.9)	2,483 (10.4)	
1–2 times/week	84,472 (25.9)	7,318 (27.2)	6,158 (28.1)	65,352 (25.8)	5,644 (23.6)	
3–4 times/week	76,020 (23.3)	7,043 (26.2)	4,974 (22.7)	59,214 (23.4)	4,789 (20.0)	
Daily or almost daily	66,922 (20.5)	5,041 (18.8)	3,490 (15.9)	53,476 (21.1)	4,915 (20.6)	
Unknown	639 (0.2)	35 (0.1)	36 (0.2)	506 (0.2)	62 (0.3)	
Physical activity, *n* (%)						<0.001
Low	46,303 (14.2)	3,090 (11.5)	2,935 (13.4)	36,399 (14.4)	3,879 (16.2)	
Moderate	102,769 (31.5)	9,117 (33.9)	7,103 (32.4)	79,547 (31.4)	7,002 (29.3)	
High	104,174 (31.9)	9,987 (37.2)	7,431 (33.9)	79,978 (31.6)	6,778 (28.4)	
Unknown	72,972 (22.4)	4,680 (17.4)	4,479 (20.4)	57,570 (22.7)	6,243 (26.1)	
Total physical activity, MET-min/week, median [IQR]	2,539.04 [1,072.00, 2,910.00]	2,507.25 [1,173.00, 3,132.00]	2,539.04 [1,087.50, 2,994.50]	2,539.04 [1,062.00, 2,892.00]	2,539.04 [1,020.00, 2,788.05]	<0.001
Body mass index, kg/m^2^, mean (SD)	27.35 (4.70)	22.33 (1.72)	26.88 (3.24)	27.77 (4.66)	28.97 (5.04)	<0.001
*Biological aging measures*						
PhenoAge, years, mean (SD)	47.82 (10.24)	40.46 (8.91)	43.41 (9.27)	48.27 (9.82)	55.39 (10.13)	<0.001
PhenoAge acceleration, years, mean (SD)	−0.00 (5.94)	−2.30 (5.04)	−0.46 (5.14)	0.05 (5.80)	2.45 (7.71)	<0.001
PhenoAge acceleration, SD units, mean (SD)	−0.00 (1.00)	−0.39 (0.85)	−0.08 (0.87)	0.01 (0.98)	0.41 (1.30)	<0.001
PhenoAge acceleration quartile, *n* (%)						<0.001
Q1	81,559 (25.0)	10,913 (40.6)	5,633 (25.7)	61,041 (24.1)	3,972 (16.6)	
Q2	81,561 (25.0)	7,344 (27.3)	5,782 (26.3)	63,645 (25.1)	4,790 (20.0)	
Q3	81,552 (25.0)	5,241 (19.5)	5,743 (26.2)	64,785 (25.6)	5,783 (24.2)	
Q4	81,546 (25.0)	3,376 (12.6)	4,790 (21.8)	64,023 (25.3)	9,357 (39.1)	
PhenoAge accelerated aging, *n* (%)						<0.001
≤0 years	182,404 (55.9)	19,652 (73.1)	12,756 (58.1)	139,987 (55.2)	10,009 (41.9)	
>0 years	143,814 (44.1)	7,222 (26.9)	9,192 (41.9)	113,507 (44.8)	13,893 (58.1)	
KDM biological age, years, mean (SD)	56.43 (8.27)	50.01 (7.60)	51.75 (7.85)	57.03 (7.96)	61.64 (6.46)	<0.001
KDM acceleration, years, mean (SD)	−0.00 (1.70)	−1.51 (1.72)	−0.85 (1.63)	0.21 (1.61)	0.24 (1.60)	<0.001
KDM acceleration, SD units, mean (SD)	−0.00 (1.00)	−0.89 (1.01)	−0.50 (0.95)	0.12 (0.95)	0.14 (0.94)	<0.001
KDM acceleration quartile, *n* (%)						<0.001
Q1	81,589 (25.0)	17,464 (65.0)	10,702 (48.8)	49,949 (19.7)	3,474 (14.5)	
Q2	81,546 (25.0)	5,257 (19.6)	5,071 (23.1)	65,610 (25.9)	5,608 (23.5)	
Q3	81,519 (25.0)	2,316 (8.6)	3,171 (14.4)	67,572 (26.7)	8,460 (35.4)	
Q4	81,564 (25.0)	1,837 (6.8)	3,004 (13.7)	70,363 (27.8)	6,360 (26.6)	
KDM accelerated aging, *n* (%)						<0.001
≤0 years	169,739 (52.0)	22,975 (85.5)	16,054 (73.1)	121,062 (47.8)	9,648 (40.4)	
>0 years	156,479 (48.0)	3,899 (14.5)	5,894 (26.9)	132,432 (52.2)	14,254 (59.6)	
*Follow-up and incident events*						
Follow-up time, years, median [IQR]	13.48 [12.74, 14.09]	13.56 [12.93, 14.13]	13.50 [12.86, 14.13]	13.49 [12.74, 14.09]	13.31 [12.44, 14.00]	<0.001
Incident death, *n* (%)						<0.001
No	299,761 (91.9)	25,999 (96.7)	21,084 (96.1)	233,833 (92.2)	18,845 (78.8)	
Yes	26,457 (8.1)	875 (3.3)	864 (3.9)	19,661 (7.8)	5,057 (21.2)	
Incident CVD, *n* (%)						<0.001
No	266,393 (81.7)	25,324 (94.2)	20,286 (92.4)	212,321 (83.8)	8,462 (35.4)	
Yes	59,825 (18.3)	1,550 (5.8)	1,662 (7.6)	41,173 (16.2)	15,440 (64.6)	
Incident diabetes, *n* (%)						<0.001
No	301,214 (92.3)	26,765 (99.6)	21,652 (98.7)	233,552 (92.1)	19,245 (80.5)	
Yes	25,004 (7.7)	109 (0.4)	296 (1.3)	19,942 (7.9)	4,657 (19.5)	
Incident CKD, *n* (%)						<0.001
No	310,369 (95.1)	26,648 (99.2)	21,610 (98.5)	241,918 (95.4)	20,193 (84.5)	
Yes	15,849 (4.9)	226 (0.8)	338 (1.5)	11,576 (4.6)	3,709 (15.5)	
Incident hypertension, *n* (%)						<0.001
No	245,863 (75.4)	26,106 (97.1)	20,871 (95.1)	183,540 (72.4)	15,346 (64.2)	
Yes	80,355 (24.6)	768 (2.9)	1,077 (4.9)	69,954 (27.6)	8,556 (35.8)	

Data are presented as mean (SD), median [IQR], or *n* (%), as appropriate. CKM stages were defined according to the American Heart Association framework, with stages 2 and 3 combined as an intermediate category in the present analysis. Biological age acceleration was defined as the residual from the regression of biological age on chronological age, and accelerated aging was defined as an acceleration value greater than 0 years. Incident events were identified during follow-up after baseline assessment. CKM, cardiovascular–kidney–metabolic; SD, standard deviation; IQR, interquartile range; MET, metabolic equivalent task; KDM, Klemera–Doubal method; CVD, cardiovascular disease; CKD, chronic kidney disease.

**Table 2.** Survey-weighted baseline characteristics of NHANES participants according to CKM stage.

Characteristic	Overall (*N* = 5,228)	Stage 0 (*N* = 516)	Stage 1 (*N* = 1,284)	Stage 2–3 (*N* = 2,923)	Stage 4 (*N* = 505)	*P* value
*Demographics and socioeconomic factors*						
Age, years, weighted mean (SD)	46.70 (16.61)	35.18 (12.54)	41.39 (14.75)	49.72 (15.78)	64.53 (13.43)	<0.001
Sex, unweighted *n* (weighted %)						<0.001
Male	2,597 (48.8)	195 (35.3)	665 (50.8)	1,443 (50.6)	294 (52.7)	
Female	2,631 (51.2)	321 (64.7)	619 (49.2)	1,480 (49.4)	211 (47.3)	
Race and ethnicity, unweighted *n* (weighted %)						0.002
Mexican American	914 (7.8)	67 (5.4)	245 (9.5)	550 (8.0)	52 (4.3)	
Other Hispanic	489 (4.6)	37 (3.3)	132 (5.7)	270 (4.5)	50 (4.2)	
Non-Hispanic White	2,448 (70.7)	251 (73.2)	535 (67.3)	1,374 (71.3)	288 (74.3)	
Non-Hispanic Black	996 (10.1)	92 (8.9)	279 (11.9)	530 (9.5)	95 (10.7)	
Other race or multiracial	381 (6.8)	69 (9.2)	93 (5.7)	199 (6.7)	20 (6.5)	
Education, unweighted *n* (weighted %)						<0.001
Less than 9th grade	582 (5.6)	28 (2.9)	91 (3.8)	379 (6.7)	84 (9.5)	
9–11th grade	733 (10.3)	62 (7.9)	168 (9.0)	426 (11.0)	77 (14.8)	
High school graduate/GED	1,267 (24.2)	105 (19.9)	293 (21.8)	734 (25.8)	135 (28.5)	
Some college or AA degree	1,469 (30.9)	147 (28.3)	396 (32.4)	796 (30.9)	130 (30.2)	
College graduate or above	1,177 (29.0)	174 (41.0)	336 (33.0)	588 (25.7)	79 (17.0)	
Marital status, unweighted *n* (weighted %)						<0.001
Married	2,839 (56.9)	238 (48.4)	665 (56.4)	1,672 (59.0)	264 (57.6)	
Widowed	409 (5.4)	7 (0.4)	43 (2.0)	254 (6.8)	105 (17.5)	
Divorced	545 (9.7)	35 (6.4)	107 (8.7)	332 (10.6)	71 (12.5)	
Separated	166 (2.4)	4 (1.0)	42 (2.2)	108 (2.9)	12 (1.9)	
Never married	827 (16.7)	162 (29.1)	288 (20.6)	348 (13.6)	29 (4.2)	
Living with partner	442 (8.9)	70 (14.7)	139 (10.1)	209 (7.2)	24 (6.3)	
Poverty income ratio, weighted median [IQR]	3.12 [1.67–5.00]	3.49 [1.83–5.00]	3.22 [1.76–5.00]	3.14 [1.67–5.00]	2.12 [1.18–3.63]	<0.001
*Lifestyle and clinical characteristics*						
Alcohol use, unweighted *n* (weighted %)						<0.001
No	1,540 (24.1)	133 (20.6)	326 (20.1)	922 (26.3)	159 (28.7)	
Yes	3,688 (75.9)	383 (79.4)	958 (79.9)	2,001 (73.7)	346 (71.3)	
Smoking status, unweighted *n* (weighted %)						<0.001
No	2,761 (51.9)	298 (58.2)	751 (56.8)	1,516 (50.3)	196 (34.6)	
Yes	2,467 (48.1)	218 (41.8)	533 (43.2)	1,407 (49.7)	309 (65.4)	
Hypertension, unweighted *n* (weighted %)						<0.001
No	3,372 (68.8)	516 (100.0)	1,284 (100.0)	1,459 (51.9)	113 (21.9)	
Yes	1,856 (31.2)	0 (0.0)	0 (0.0)	1,464 (48.1)	392 (78.1)	
Diabetes, unweighted *n* (weighted %)						<0.001
No	4,565 (91.3)	516 (100.0)	1,284 (100.0)	2,439 (87.6)	326 (70.9)	
Yes	663 (8.7)	0 (0.0)	0 (0.0)	484 (12.4)	179 (29.1)	
*Biological aging measures*						
PhenoAge, years, weighted mean (SD)	59.19 (18.88)	43.47 (12.95)	52.71 (15.43)	63.14 (17.61)	81.84 (15.97)	<0.001
PhenoAge acceleration, years, weighted mean (SD)	−0.11 (6.95)	−3.62 (4.82)	−0.95 (5.51)	0.65 (7.37)	3.66 (8.57)	<0.001
PhenoAge accelerated aging, unweighted *n* (weighted %)						<0.001
≤0 years	2,835 (57.9)	394 (78.4)	794 (63.8)	1,460 (52.6)	187 (39.0)	
>0 years	2,393 (42.1)	122 (21.6)	490 (36.2)	1,463 (47.4)	318 (61.0)	
KDM biological age, years, weighted mean (SD)	35.73 (23.91)	26.32 (21.88)	29.46 (22.41)	38.76 (23.74)	53.36 (19.37)	<0.001
KDM acceleration, years, weighted mean (SD)	−0.81 (16.43)	1.55 (17.82)	−1.66 (17.22)	−0.87 (15.86)	−1.39 (14.61)	0.035
KDM accelerated aging, unweighted *n* (weighted %)						<0.001
≤0 years	2,741 (52.1)	217 (40.6)	699 (53.9)	1,532 (53.5)	293 (55.5)	
>0 years	2,487 (47.9)	299 (59.4)	585 (46.1)	1,391 (46.5)	212 (44.5)	

Data are presented as survey-weighted mean (SD), weighted median [IQR], or unweighted *n* (weighted %), as appropriate. *P* values account for survey weights, strata and primary sampling units. The pooled fasting subsample weight was WTSAF2YR/3. CKM stages 2 and 3 were combined. Biological age acceleration was defined as the residual from the regression of biological age on chronological age, and accelerated aging was defined as an acceleration value greater than 0 years. CKM stages represent baseline stage burden rather than longitudinal progression. CKM, cardiovascular–kidney–metabolic; NHANES, National Health and Nutrition Examination Survey; SD, standard deviation; IQR, interquartile range; KDM, Klemera–Doubal method; PIR, Poverty Income Ratio; CVD, cardiovascular disease; CKD, chronic kidney disease.

### Biological aging measures and CKM burden or progression

In the UK Biobank, the median follow-up time was 13.48 years (IQR, 12.74–14.09). For progression from CKM stage 0/1 to the combined stage 2/3 category, 48,822 participants were included in the risk set; 2,567 experienced progression and 1,456 died before progression. For progression from stage 1 or the combined stage 2/3 category to stage 4, 275,442 participants were included; 42,835 experienced progression, and 12,814 died before progression (Table 3). In the UK Biobank, higher biological age acceleration was consistently associated with increased risks of CKM progression. After full adjustment, each 1-SD increase in PhenoAge acceleration was associated with progression from CKM stage 0/1 to stage 2/3 (cause-specific HR 1.234, 95% CI 1.189–1.281; *P* < 0.001) and from stage 1 or 2/3 to stage 4 (HR 1.208, 95% CI 1.197–1.218; *P* < 0.001). The corresponding HRs for KDMAge acceleration were 1.229 (95% CI 1.180–1.280; *P* < 0.001) and 1.220 (95% CI 1.207–1.232; *P* < 0.001) (Tables 3 and S9). Fine–Gray analyses treating death before progression as a competing event yielded corresponding sHRs of 1.214 (95% CI 1.173–1.257; *P* < 0.001) and 1.184 (95% CI 1.174–1.195; *P* < 0.001) for PhenoAge acceleration, and 1.218 (95% CI 1.171–1.266; *P* < 0.001) and 1.210 (95% CI 1.196–1.223; *P* < 0.001) for KDMAge acceleration (Tables 3 and S10). For progression to CKM stage 4, a cross-check with cmprsk::crr reproduced the primary sHR estimates and 95% CIs to three decimal places (Table S10). All primary Cox associations remained significant after false discovery rate correction (all adjusted *P* values <0.001; Table S11).

**Table 3.** Associations of biological age acceleration with CKM progression in UK Biobank and survey-weighted CKM stage burden in NHANES.

Cohort	Outcome	Exposure	*N*/events/deaths or cases/reference	Per increment	Accelerated aging >0 vs ≤0 years	Q2 vs Q1	Q3 vs Q1	Q4 vs Q1	*P* trend
UK Biobank	CKM stage 0/1 to stage 2/3	PhenoAge acceleration	48,822/2,567/1,456	HR 1.234 (1.189–1.281); *P* < 0.001 sHR 1.214 (1.173–1.257); *P* < 0.001	1.475 (1.360–1.598); *P* < 0.001	1.254 (1.126–1.398); *P* < 0.001	1.507 (1.351–1.681); *P* < 0.001	1.845 (1.646–2.067); *P* < 0.001	<0.001
UK Biobank	CKM stage 0/1 to stage 2/3	KDMAge acceleration	48,822/2,567/1,456	HR 1.229 (1.180–1.280); *P* < 0.001 sHR 1.218 (1.171–1.266); *P* < 0.001	1.480 (1.350–1.623); *P* < 0.001	1.251 (1.107–1.414); *P* < 0.001	1.504 (1.317–1.716); *P* < 0.001	1.746 (1.545–1.974); *P* < 0.001	<0.001
UK Biobank	CKM stage 1 or 2/3 to stage 4	PhenoAge acceleration	275,442/42,835/12,814	HR 1.208 (1.197–1.218); *P* < 0.001 sHR 1.184 (1.174–1.195); *P* < 0.001	1.319 (1.293–1.345); *P* < 0.001	1.095 (1.063–1.127); *P* < 0.001	1.204 (1.170–1.239); *P* < 0.001	1.552 (1.509–1.596); *P* < 0.001	<0.001
UK Biobank	CKM stage 1 or 2/3 to stage 4	KDMAge acceleration	275,442/42,835/12,814	HR 1.220 (1.207–1.232); *P* < 0.001 sHR 1.210 (1.196–1.223); *P* < 0.001	1.374 (1.348–1.401); *P* < 0.001	1.002 (0.967–1.039); *P* = 0.898	1.219 (1.177–1.262); *P* < 0.001	1.600 (1.551–1.650); *P* < 0.001	<0.001
NHANES	CKM stage 1 vs stage 0	PhenoAge acceleration	1,800/1,284/516	1.154 (1.107–1.202); *P* < 0.001	–	–	–	–	–
NHANES	CKM stage 1 vs stage 0	KDMAge acceleration	1,800/1,284/516	1.025 (1.006–1.044); *P* = 0.012	–	–	–	–	–
NHANES	CKM stage 2/3 vs stage 0	PhenoAge acceleration	3,439/2,923/516	1.221 (1.176–1.268); *P* < 0.001	–	–	–	–	–
NHANES	CKM stage 2/3 vs stage 0	KDMAge acceleration	3,439/2,923/516	1.038 (1.019–1.057); *P* < 0.001	–	–	–	–	–
NHANES	CKM stage 4 vs stage 0	PhenoAge acceleration	1,021/505/516	1.333 (1.249–1.423); *P* < 0.001	–	–	–	–	–
NHANES	CKM stage 4 vs stage 0	KDMAge acceleration	1,021/505/516	1.041 (1.011–1.073); *P* = 0.010	–	–	–	–	–

For UK Biobank, per-increment estimates are cause-specific HRs from Cox proportional hazards models and subdistribution hazard ratios (sHRs) from Fine–Gray competing-risk models, with death before CKM progression treated as a competing event. All estimates are presented with 95% CIs. Analyses assessed transitions from stage 0/1 to combined stage 2/3 and from stage 1 or 2/3 to stage 4. The *N*/events/deaths or cases/reference column denotes *N*/progression events/deaths before progression in UK Biobank and unweighted *N*/cases/stage 0 reference in NHANES. Fine–Gray sHRs for both transitions were estimated using the full transition-specific risk sets with participant-clustered robust variance. NHANES estimates are survey-weighted ORs with 95% CIs from stage-specific logistic regression models comparing each CKM stage with stage 0. Analyses applied the pooled fasting subsample weight WTSAF2YR/3 and accounted for SDMVSTRA and SDMVPSU using Taylor series linearization; Model 3 additionally adjusted for survey cycle. Absolute PhenoAge and KDMAge and their acceleration measures were modeled separately in continuous NHANES analyses. Because acceleration was defined as the residual from regressing biological age on chronological age, identical samples, covariates, and linear adjustment for chronological age yielded algebraically equivalent per-year exposure coefficients. This table therefore presents only continuous acceleration estimates. All estimates are from the fully adjusted Model 3. In UK Biobank, biological age acceleration was analyzed per 1-SD increment, as accelerated aging (>0 vs ≤0 years), and by quartiles using Q1 as the reference. In NHANES, biological age acceleration was analyzed per 1-year increment. *P* trend was calculated across quartiles in UK Biobank. CKM, cardiovascular–kidney–metabolic; NHANES, National Health and Nutrition Examination Survey; HR, cause-specific hazard ratio; sHR, subdistribution hazard ratio; OR, odds ratio; CI, confidence interval; KDM, Klemera–Doubal method.

When biological age acceleration was modeled categorically, participants with accelerated aging had higher risks of CKM progression than those without accelerated aging. For progression from stage 0/1 to the combined stage 2/3 category, the HRs were 1.475 (95% CI 1.360–1.598; *P* < 0.001) for PhenoAge acceleration and 1.480 (95% CI 1.350–1.623; *P* < 0.001) for KDMAge acceleration. For progression to stage 4, the corresponding HRs were 1.319 (95% CI 1.293–1.345; *P* < 0.001) and 1.374 (95% CI 1.348–1.401; *P* < 0.001), respectively. In quartile-based analyses, participants in the highest quartile of PhenoAge acceleration had HRs of 1.845 (95% CI 1.646–2.067; *P* < 0.001) and 1.552 (95% CI 1.509–1.596; *P* < 0.001) for the two progression outcomes, respectively. The corresponding HRs for the highest quartile of KDMAge acceleration were 1.746 (95% CI 1.545–1.974; *P* < 0.001) and 1.600 (95% CI 1.551–1.650; *P* < 0.001) (Figure 2 and Tables 3, S12, and S13).

**Figure 2.**
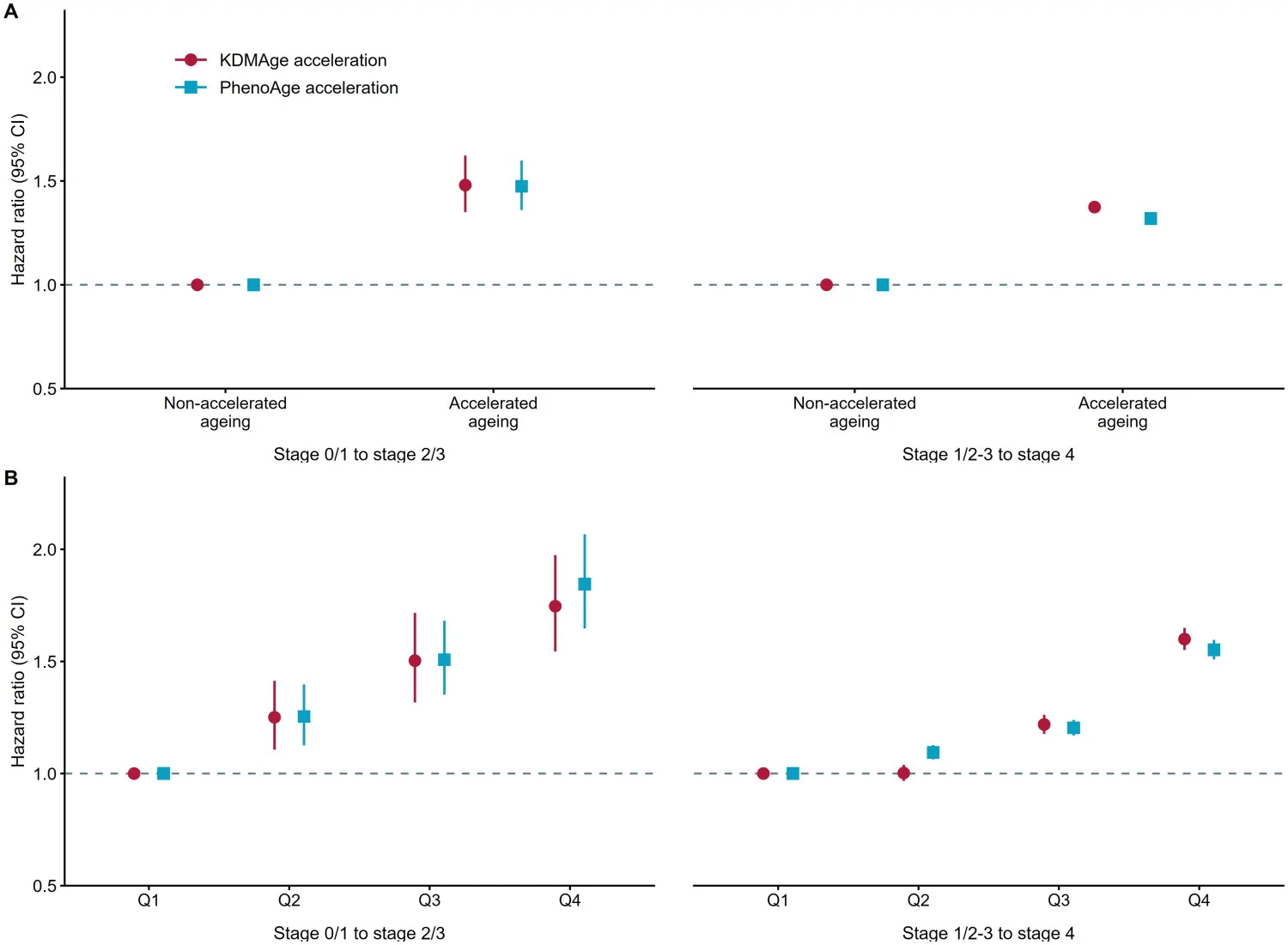
Associations of categorical biological age acceleration with CKM progression in the UK Biobank. HRs and 95% CIs were estimated using Cox proportional hazards models in the UK Biobank. Panel A shows the associations of accelerated aging status, defined as biological age acceleration >0 vs ≤0 years, with CKM progression. Panel B shows the associations of quartiles of biological age acceleration with CKM progression, using Q1 as the reference category. Two CKM progression outcomes were evaluated: progression from CKM stage 0/1 to the combined stage 2/3 category, and progression from stage 1 or the combined stage 2/3 category to stage 4. Models were fully adjusted according to Model 3. CKM, cardiovascular–kidney–metabolic; KDM, Klemera–Doubal method.

In survey-weighted NHANES analyses additionally adjusted for survey cycle, each 1-year increase in PhenoAge acceleration was associated with higher odds of CKM stage 1 (OR 1.154, 95% CI 1.107–1.202; *P* < 0.001), stage 2/3 (OR 1.221, 95% CI 1.176–1.268; *P* < 0.001), and stage 4 (OR 1.333, 95% CI 1.249–1.423; *P* < 0.001) relative to stage 0. KDMAge acceleration was likewise associated with stage 1 (OR 1.025, 95% CI 1.006–1.044; *P* = 0.012), stage 2/3 (OR 1.038, 95% CI 1.019–1.057; *P* < 0.001), and stage 4 (OR 1.041, 95% CI 1.011–1.073; *P* = 0.010) (Tables 3 and S14). All six associations remained significant after false discovery rate correction (Table S15). In *post hoc* diagnostic models, KDMAge acceleration ORs changed from below 1 with adjustment for age alone to above 1 after sex was added (Table S16). The positive KDMAge associations persisted when age was modeled using quadratic or spline terms. Absolute biological age and age acceleration were modeled separately; Table S14 presents both parameterizations, whereas the main text and Table 3 report acceleration estimates.

Sensitivity analyses yielded similar results. After excluding participants who experienced CKM progression within the first year of follow-up, the fully adjusted HRs per 1-SD increment in PhenoAge acceleration were 1.238 (95% CI, 1.193–1.286) for progression to stage 2/3 and 1.203 (95% CI, 1.193–1.214) for progression to stage 4. The corresponding HRs for KDMAge acceleration were 1.231 (95% CI, 1.182–1.283) and 1.216 (95% CI, 1.203–1.229). In complete-case analyses for covariates, the associations remained consistent, with HRs of 1.240 (95% CI, 1.188–1.294) and 1.203 (95% CI, 1.191–1.215) for PhenoAge acceleration, and 1.243 (95% CI, 1.185–1.304) and 1.224 (95% CI, 1.209–1.240) for KDMAge acceleration, respectively (Tables S17 and S18). The proportional hazards assumption was evaluated using Schoenfeld residuals. No evidence of violation was observed for progression from CKM stage 0/1 to the combined stage 2/3 category. For progression to stage 4, Schoenfeld residual tests suggested time-varying effects for both PhenoAge acceleration and KDMAge acceleration. Therefore, time-stratified Cox analyses were performed across follow-up intervals of 0–5, 5–10, and >10 years. The associations remained directionally consistent across all intervals, with HRs above 1 for both biological age acceleration measures, although the magnitude of association was slightly stronger during earlier follow-up (Tables S19 and S20).

In NHANES, the multiple-imputation analysis included 6,031 participants, compared with 5,228 in the survey-weighted complete-case analysis. Across the six fully adjusted biological age acceleration associations, no changes in direction or statistical significance were observed. All six ORs changed by less than 5%, and the largest absolute change was 1.2% (Table S21).

### Restricted cubic spline analyses

In the UK Biobank, restricted cubic spline analyses showed significant overall associations between biological age acceleration and CKM progression. For progression from CKM stage 0/1 to the combined stage 2/3 category, PhenoAge acceleration was associated with CKM progression with evidence of non-linearity (*P* overall <0.001; *P* nonlinear = 0.009). By contrast, KDMAge acceleration showed a significant overall association with this transition, but without clear evidence of non-linearity (*P* overall <0.001; *P* nonlinear = 0.334). For progression from stage 1 or the combined stage 2/3 category to stage 4, both PhenoAge acceleration (*P* overall <0.001; *P* nonlinear = 0.002) and KDMAge acceleration (*P* overall <0.001; *P* nonlinear <0.001) showed significant overall and non-linear associations with CKM progression (Figure 3 and Table S22). The conclusions regarding non-linearity were unchanged across alternative knot specifications, although the four-knot KDMAge model for progression to stage 4 showed greater lower-tail curvature. The near-null Q2-versus-Q1 estimate in the corresponding quartile analysis was consistent with the relatively flat association at the lower end of the curve. No clinically validated threshold or biological cutoff was identified (Table S23 and Figure S1).

**Figure 3.**
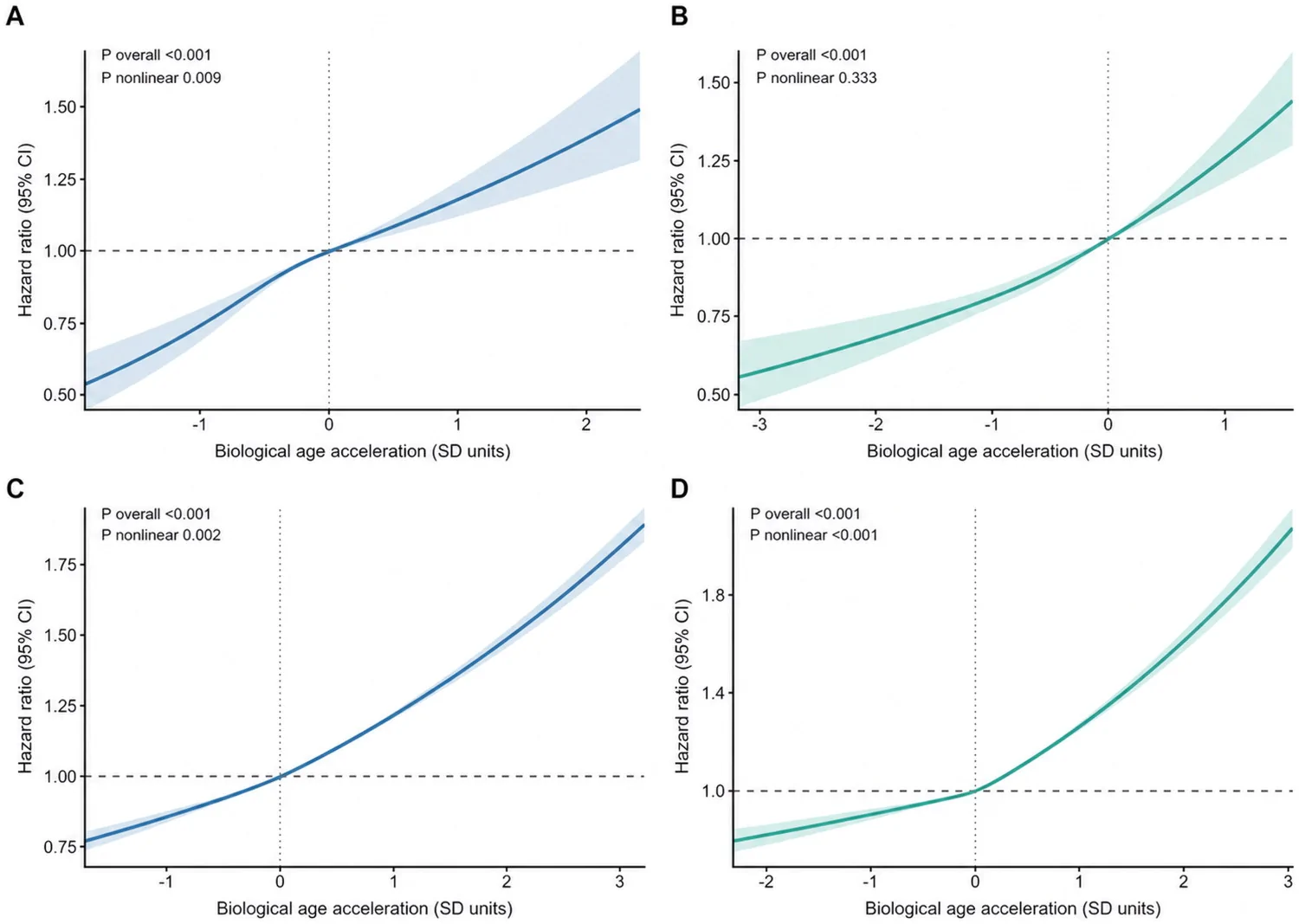
Dose-response associations between biological age acceleration and CKM progression in the UK Biobank. Restricted cubic spline curves show the associations of PhenoAge acceleration and KDMAge acceleration with CKM progression in the UK Biobank. HRs and 95% CIs were estimated using fully adjusted Cox proportional hazards models, with standardized biological age acceleration modeled as a continuous exposure. The reference value was 0 SD, and knots were placed at the 25th, 50th, and 75th percentiles. Two progression outcomes were assessed: progression from CKM stage 0/1 to the combined stage 2/3 category, and progression from stage 1 or the combined stage 2/3 category to stage 4. Models were fully adjusted according to Model 3. CKM, cardiovascular–kidney–metabolic; KDM, Klemera–Doubal method.

### Subgroup analyses

In the UK Biobank, the positive associations of PhenoAge acceleration and KDMAge acceleration with CKM progression were largely consistent across predefined subgroups. For progression from CKM stage 0/1 to the combined stage 2/3 category, associations remained directionally similar after stratification by sex, age, ethnicity, and baseline CKM stage. No significant interactions were detected, indicating limited evidence of effect heterogeneity for early CKM progression.

For progression from CKM stage 1 or the combined stage 2/3 category to stage 4, the association between PhenoAge acceleration and CKM progression appeared slightly stronger in women than in men, with a significant interaction by sex. A stronger association was also observed among participants aged <60 years than among those aged ≥60 years, with evidence of interaction by age group. The association was additionally more pronounced among participants with baseline CKM stage 2/3 than among those with baseline stage 1. KDMAge acceleration showed a similar positive association with progression to stage 4, although effect modification was mainly observed by age group, ethnicity, and baseline CKM stage. Higher risk estimates were noted among participants aged <60 years, non-white participants, and those with baseline CKM stage 2/3 (Figure 4 and Tables S24 and S25).

**Figure 4.**
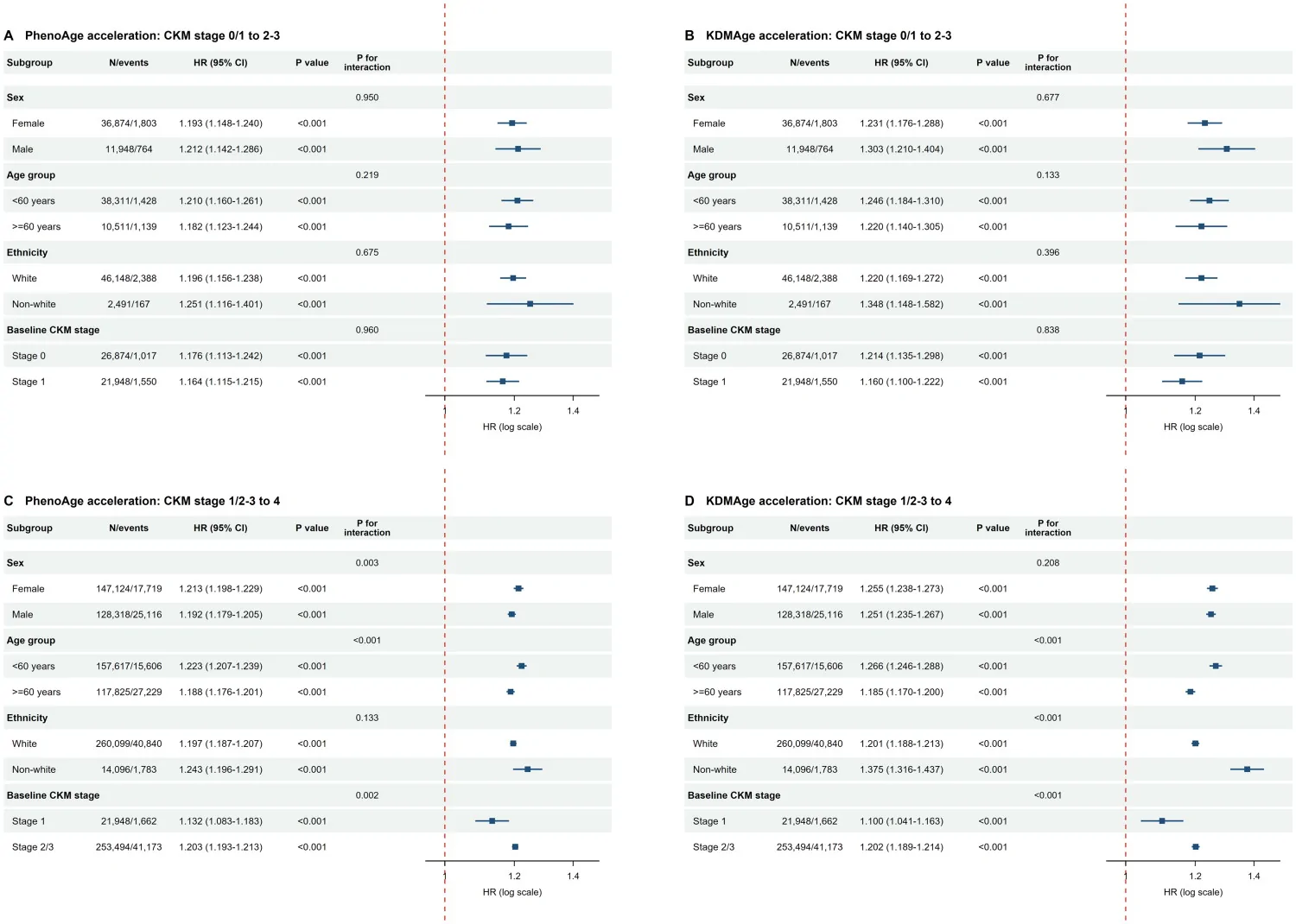
Subgroup analyses of biological age acceleration and CKM progression in the UK Biobank. Forest plots show the associations of PhenoAge acceleration and KDMAge acceleration with CKM progression across predefined subgroups in the UK Biobank. HRs and 95% CIs were estimated per 1-SD increment in biological age acceleration using Cox proportional hazards models. Subgroups included sex, age group, ethnicity and baseline CKM stage. Two CKM progression outcomes were evaluated: progression from CKM stage 0/1 to the combined stage 2/3 category, and progression from stage 1 or the combined stage 2/3 category to stage 4. *P* values for interaction were used to assess heterogeneity of associations across subgroups. CKM, cardiovascular–kidney–metabolic; KDM, Klemera–Doubal method.

## Discussion

This study investigated the associations of biological age acceleration with CKM progression in the UK Biobank and with baseline CKM stage burden in NHANES. In the UK Biobank, greater PhenoAge acceleration and KDMAge acceleration were associated with higher risks of subsequent CKM progression across two prespecified stage transitions. Survey-weighted analyses in NHANES showed positive cross-sectional associations between both biological aging measures and more advanced baseline CKM stage burden. Restricted cubic spline analyses in the UK Biobank further indicated potential non-linear relationships. Collectively, these findings support the relevance of blood chemistry-based biological aging measures to CKM outcomes, although incremental predictive performance and clinical utility were not evaluated.

Previous work relating biological aging to renal and cardiometabolic outcomes has often focused on individual aging markers, most notably leukocyte telomere length (LTL). Shorter LTL has been associated with increased risks of CVD, CKD, and mortality in population-based and disease-specific settings [14–16]. Among patients with CKD, relative telomere length has also been related to CVD burden and subsequent cardiovascular outcomes [14]. Nevertheless, evidence linking LTL to kidney function decline or CKD progression has been less consistent, suggesting that a single aging biomarker may not sufficiently reflect the multisystem perturbations that characterize CKM syndrome [17,18]. As CKM syndrome encompasses interrelated metabolic, inflammatory, renal, and cardiovascular abnormalities, composite indices may be better suited to capturing biological aging across multiple physiological domains [2]. In contrast to single biomarkers, blood chemistry-based aging measures, including PhenoAge and KDMAge, integrate signals related to inflammation, metabolism, immune status, and organ function. These indices have been associated with several chronic disease outcomes, including chronic respiratory diseases and rheumatoid arthritis, in large observational studies [19,20].

PhenoAge integrates inflammatory and hematological markers, including C-reactive protein, white blood cell count, and lymphocyte percentage, and may therefore index systemic inflammation, immune dysregulation, and metabolic stress [21]. These processes are closely linked to endothelial dysfunction, renal injury, and cardiovascular remodeling, which are central features of CKM pathophysiology [2]. By contrast, KDMAge incorporates physiological and biochemical indicators related to pulmonary function, blood pressure regulation, metabolic status, kidney function, and systemic inflammation, thereby reflecting a broader disturbance of physiological homeostasis [10,22]. Previous studies linking clinical biomarker-based measures of biological aging to CKD, cancer, depression, and anxiety further support their relevance to multisystem disease processes beyond chronological age [13,23,24].

In the UK Biobank, both PhenoAge acceleration and KDMAge acceleration were associated with higher risks of the two prespecified CKM progression outcomes. Associations also remained positive in competing-risk analyses treating death before progression as a competing event. This finding is consistent with the biological basis of CKM progression, in which metabolic, renal, inflammatory, and cardiovascular systems deteriorate in a coordinated manner rather than through a single biomarker pathway [25]. Across alternative knot specifications, the non-linear inference pattern was unchanged, although the four-knot KDMAge model for progression to stage 4 showed greater lower-tail curvature. This local variation may reflect model specification and should not be interpreted as evidence of a clinically meaningful threshold or biological cutoff. After incorporating NHANES sampling weights, strata, primary sampling units, and survey cycle, all six continuous age acceleration associations remained positive and statistically significant, with the largest PhenoAge estimate observed for stage 4. The algebraically equivalent continuous absolute-age and age acceleration parameterizations were not interpreted as independent findings. *Post hoc* diagnostic findings were consistent with sex composition contributing to the discordant NHANES KDMAge patterns. The smaller per-year KDMAge ORs may partly reflect its wider acceleration distribution (SD 16.4 versus 7.0 years for PhenoAge), together with differences in biomarker composition and overlap with CKM staging. These cross-sectional data cannot distinguish measurement effects from biological differences, so the effect-size contrast should not be interpreted as evidence of differential clinical importance.

The findings of this study should be interpreted within the CKM staging framework, which was developed to characterize the interrelated progression of adiposity, metabolic dysfunction, CKD, and CVD, rather than considering these conditions as isolated states [26]. This framework is clinically relevant because cardiometabolic risk factors and impaired kidney function may interact over time, jointly increasing vascular injury, cardiovascular events, and mortality risk [4,27]. In the present analysis, stages 2 and 3 were combined because standardized information required to define subclinical CVD was not consistently available in both the UK Biobank and NHANES, and cohort-specific surrogate definitions could have increased misclassification. Accordingly, two clinically interpretable CKM transitions were examined in the UK Biobank: progression from stage 0/1 to the combined stage 2/3 category, reflecting the emergence of metabolic risk factors, CKD, or intermediate CKM burden; and progression from stage 1 or stage 2/3 to stage 4, indicating overt clinical CVD, a major adverse endpoint emphasized in CKM risk assessment [3].

Both PhenoAge acceleration and KDMAge acceleration were associated with these two CKM transitions in the present study, suggesting that biological aging may be relevant across different phases of CKM deterioration. This pattern is biologically plausible because CKM syndrome is increasingly understood as an integrated cardio-renal-metabolic condition in which metabolic dysfunction, kidney impairment, inflammation, and cardiovascular injury may reinforce one another [28,29]. From an aging perspective, composite biomarker profiles can capture systemic physiological dysregulation and preclinical vulnerability across multiple organ systems [30,31]. These findings do not establish biological aging measures as standalone screening tools. Rather, future studies should formally evaluate whether these measures improve discrimination, calibration, and clinical decision-making beyond established CKM risk factors [32].

This study has several strengths. Firstly, the UK Biobank provided a large prospective cohort for evaluating incident CKM progression, whereas NHANES provided an independent population-based cross-sectional assessment of baseline CKM stage burden. Secondly, two biological age algorithms, PhenoAge and KDMAge, were used to derive their respective acceleration measures and evaluated in parallel. Thirdly, competing-risk analyses in UK Biobank accounted for death before CKM progression, whereas survey-weighted NHANES models incorporated fasting subsample weights, strata, primary sampling units, and survey cycle. Multiple-imputation analyses examined the influence of incomplete observed data, while UK Biobank sensitivity, restricted cubic spline, and subgroup analyses assessed the robustness and shape of the longitudinal associations.

Several limitations should be acknowledged. Firstly, biological age was measured only at baseline; therefore, temporal changes in biological aging and their relationship with CKM progression could not be assessed. Secondly, although the UK Biobank analyses were longitudinal, the observational design does not permit causal inference, and reverse causation cannot be fully ruled out for baseline associations. Thirdly, residual confounding may persist; although physical activity was included in the UK Biobank models, comparable physical activity, and broader lifestyle-medicine domains, including diet, sleep, sedentary behavior, psychosocial stress, medication dose or duration, genetic background, and longitudinal inflammatory burden, were not fully incorporated across both cohorts. Fourthly, participants with missing biomarkers required for biological age estimation were excluded, which may have introduced selection bias despite comparisons between included and excluded participants and sensitivity analyses. In NHANES, analytic-sample inclusion was associated with observed characteristics and did not support a missing-completely-at-random mechanism. Although multiple imputation produced similar results, it depended on an unverifiable conditional missing-at-random assumption. Participant-level imputation could not recover the two entire source cycles lacking biological age model data, leaving possible selection bias and limited temporal representativeness. Fifthly, CKM stages 2 and 3 were combined because harmonized information on subclinical CVD was not consistently available across cohorts, limiting assessment of heterogeneity within intermediate CKM stages. Sixthly, this study did not separately evaluate individual CKM components, so component-specific heterogeneity could not be assessed. Seventhly, only two blood chemistry-based aging indices were examined; whether similar findings would be observed using epigenetic clocks, telomere length or frailty-based measures remains uncertain. Eighthly, formal mediation analyses were not performed, as several candidate mediators were embedded in the biological aging algorithms or CKM stage definitions, limiting causal interpretability. The precomputed NHANES KDMAge values could not be independently verified for biomarker composition or sex-specific calibration against the UK Biobank implementation. This uncertainty limits cross-cohort comparability. Finally, shared biomarkers may induce mathematical coupling and inflate associations, particularly in NHANES. Excluding these inputs would alter the validated algorithms, precluding independent estimation. The study also did not evaluate clinical thresholds or compare models with and without biological aging measures using the C-index, calibration, net reclassification improvement, integrated discrimination improvement, or decision-curve analysis. Accordingly, these findings do not establish incremental predictive performance or clinical utility beyond established CKM risk factors.

## Conclusion

In conclusion, biological age acceleration was associated with subsequent CKM progression in the UK Biobank and with greater baseline CKM stage burden in survey-weighted NHANES analyses. These associations were observed for both PhenoAge and KDMAge acceleration, although cross-cohort comparability of KDMAge remains uncertain. UK Biobank spline analyses suggested potential non-linear patterns. These measures may warrant further investigation as potential complementary markers, but the present findings do not establish incremental predictive or clinical utility.

## Supplementary Material

Supplementary methods.docx

Supplementary Figure S1.tif

## Data Availability

All data used in this study were obtained from the UK Biobank (application number 778815) and the NHANES database. Access to the datasets is available upon request through the UK Biobank portal (https://www.ukbiobank.ac.uk/) and the NHANES website (https://wwwn.cdc.gov/nchs/nhanes/). The supplementary table data have been uploaded to the Mendeley Data repository and are available at: https://data.mendeley.com/datasets/c7hxtbtzmh/1.
