# Supplementary material for: Biological age acceleration and cardiovascular–kidney–metabolic syndrome progression: evidence from UK Biobank and NHANES
Source: Ren Fail. 2026 Oct 11;48(1):2738248. doi: 10.1080/0886022X.2026.2738248 (PMC13660544; doi:10.1080/0886022X.2026.2738248)
Supplement: Supplementary file 1 [file IRNF_A_2738248_SM2285.docx]

**Supplementary Method 1: Calculation of biological age and age acceleration**

**1. Phenotypic age (PhenoAge)**

PhenoAge was developed from mortality-based modelling in NHANES III and combines chronological age with nine routinely measured biomarkers: albumin, alkaline phosphatase (ALP), creatinine, C-reactive protein (CRP), glucose, mean corpuscular volume (MCV), red cell distribution width (RDW), white blood cell count (WBC), and lymphocyte percentage [1-4]. Biomarker units were harmonised to the requirements of the original algorithm before calculation. The PhenoAge linear predictor was calculated as follows:

xb = -19.907 - 0.0336 * albumin + 0.0095 * creatinine + 0.1953 * glucose + 0.0954 * ln(CRP) - 0.0120 * lymphocyte percentage + 0.0268 * MCV + 0.3306 * RDW + 0.00188 * ALP + 0.0554 * WBC + 0.0804 * chronological age

gamma = 0.0076927

mortality risk = 1 - exp{-exp(xb) * [exp(120 * gamma) - 1] / gamma}

PhenoAge = 141.50225 + ln[-0.00553 * ln(1 - mortality risk)] / 0.090165

**2. Klemera-Doubal method biological age (KDMAge)**

KDMAge was estimated using cohort-specific Klemera-Doubal implementations based on the biomarker panels available in each cohort. In the UK Biobank, the NC/Lancet-style implementation included forced expiratory volume in 1 s (FEV1), systolic blood pressure (SBP), albumin, alkaline phosphatase (ALP), blood urea nitrogen (BUN), creatinine, C-reactive protein (CRP), glycated haemoglobin (HbA1c), and total cholesterol. Creatinine and CRP were natural-log transformed, and biomarker units were harmonised before modelling. The NHANES validation dataset contained precomputed KDM and KDM acceleration estimates; the retained source did not permit independent verification that its full input panel was identical to the UK Biobank implementation.

For each biomarker i, the association between biomarker value and chronological age was modelled as:

x_i = q_i + k_i * CA + e_i

where x_i is the biomarker value, CA is chronological age, q_i and k_i are the fitted intercept and slope, and s_i is the residual standard error.

KDMAge was then calculated as:

KDMAge = [sum_i((x_i - q_i) * k_i / s_i^2) + CA / s_BA^2] / [sum_i(k_i^2 / s_i^2) + 1 / s_BA^2]

where s_BA^2 represents the variance parameter for biological age. For the UK Biobank implementation, biomarker-age regressions were fitted separately by sex within the complete biological-age analysis sample.

**3. Age acceleration**

PhenoAge acceleration (PAAC) and KDMAge acceleration (KDMAC) were defined as residuals from cohort-specific linear regression models in which PhenoAge and KDMAge were separately regressed on chronological age. Residuals greater than 0 indicated accelerated biological ageing, whereas residuals less than or equal to 0 indicated non-accelerated ageing.

**Supplementary Method 2: Definition of the Syndrome**

**1. Cardiovascular–Kidney–Metabolic Syndrome (CKM)**

**1.1 Preliminary Concepts**

**1) Metabolic Syndrome (MetS)**

Presence of at least three of the following:
① Waist circumference ≥88 cm in women or ≥102 cm in men (for individuals of Asian ancestry: ≥80 cm in women, ≥90 cm in men);
② High-density lipoprotein cholesterol (HDL-C) <40 mg/dL in men or <50 mg/dL in women;
③ Triglycerides ≥150 mg/dL;
④ Elevated blood pressure (systolic blood pressure ≥130 mmHg and/or diastolic blood pressure ≥80 mmHg and/or use of antihypertensive medication);
⑤ Fasting plasma glucose ≥100 mg/dL.

**2) Metabolic Risk Factors**

Presence of at least one of the following:
① Hypertension;
② Hypertriglyceridemia (≥135 mg/dL);
③ Metabolic syndrome (MetS);
④ Diabetes.

**3) Overweight/Obesity, Abdominal Obesity, or Adipose Tissue Dysfunction**

Presence of at least one of the following:
① Body mass index (BMI) ≥25 kg/m² (for Asians: ≥23 kg/m²);
② Waist circumference ≥88 cm in women / ≥102 cm in men (for Asians: ≥80 cm in women / ≥90 cm in men);
③ Fasting plasma glucose 100–124 mg/dL or HbA1c in the range of 5.7%–6.4%.

**4) Clinical Cardiovascular Disease (CVD)**

Presence of at least one of the following:
① Coronary heart disease (CHD);
② Heart failure (HF);
③ Stroke;
④ Peripheral artery disease (PAD);
⑤ Atrial fibrillation (AFib).

**1.2 CKM Classification**

CKM classification is shown in Table 1.

| CKM Stage | CKM Features | CKM Definition |
| --- | --- | --- |
| Stage 0 | No CKM risk factors | No overweight/obesity; No metabolic risk factors; No chronic kidney disease (CKD); No clinical CVD or its subtypes. |
| Stage 1 | Excess and/or dysfunctional adiposity | Presence of overweight or obesity; No other metabolic risk factors; No CKD. |
| Stage 2–3 | Metabolic risk and CKD | Presence of metabolic risk factors or CKD. |
| Stage 4 | Clinical CVD within CKM | Stage 4a: (1) Presence of Stage 1 CKM or other metabolic risk factors or CKD; (2) Presence of clinical CVD; (3) No kidney failure. Stage 4b: (1) Presence of Stage 1 CKM or other metabolic risk factors or CKD; (2) Presence of clinical CVD; (3) Presence of kidney failure. |

**Supplementary Method 3: Additional statistical analyses**

**1. NHANES complex survey design**

The NHANES validation analysis included the three nonconsecutive survey cycles represented in the biological-age dataset (2005–2006, 2007–2008, and 2015–2016). Because fasting glucose and triglycerides were components of CKM staging, analyses applied the 2-year fasting subsample weight (WTSAF2YR) divided by three. Survey-weighted stage-specific logistic regression models compared CKM stages 1, 2/3, and 4 with stage 0. Variance estimation accounted for masked strata (SDMVSTRA) and primary sampling units (SDMVPSU) using Taylor series linearization. The fully adjusted model included chronological age, sex, race and ethnicity, Poverty Income Ratio, educational attainment, marital status, smoking, alcohol use, and survey cycle. Findings were interpreted as cross-sectional associations with baseline CKM stage rather than evidence of progression.

**2. Competing-risk analysis in the UK Biobank**

Death before CKM progression was treated as a competing event in Fine–Gray proportional subdistribution hazards models adjusted for the same Model 3 covariates as the corresponding Cox models. Target progression was coded as event 1, death before progression as event 2, and all remaining observations as censored; when both occurred on the same day, progression was prioritised. For both transitions, full transition-specific risk sets were constructed using the finegray function in the survival package (version 3.8-6), followed by weighted Cox regression with participant-clustered robust variance estimation. Corresponding Cox models used the same risk sets and covariates, with death treated as a censoring event.

**3. Restricted cubic spline knot sensitivity**

The primary UK Biobank Cox models incorporating restricted cubic splines placed knots at the 25th, 50th, and 75th percentiles of standardized biological age acceleration. Sensitivity to knot placement was assessed by refitting the fully adjusted models with three knots at the 10th, 50th, and 90th percentiles or four knots at the 5th, 35th, 65th, and 95th percentiles. Overall associations were evaluated by likelihood-ratio comparisons of the spline and covariate-only models, whereas nonlinearity was assessed by comparison with the corresponding linear-exposure model. Curves used 0 SD as the reference. Alternative-knot analyses used Cox rather than Fine-Gray spline models.

**4. NHANES missing-data mechanism and multiple imputation**

Analytic-sample inclusion was first examined among participants with available CKM diagnostic source data by comparing inclusion rates across survey cycles. Survey-weighted logistic regression was then conducted within cycles represented in the biological-age dataset, with inclusion as the binary outcome and age, sex, race and ethnicity, survey cycle, and observed histories of hypertension and diabetes as predictors. Models incorporated strata, primary sampling units, and pooled examination weights. A second model restricted the denominator to participants with available biological-age model data. These analyses assessed associations between observed characteristics and sample inclusion; they could identify departures from a missing-completely-at-random mechanism but could not establish missing at random.

As a sensitivity analysis, multiple imputation by chained equations was performed under a conditional missing-at-random assumption among 6,031 participants with positive fasting subsample weights. Missing CKM stage, education, marital status, Poverty Income Ratio, and alcohol use were imputed using 30 imputations and 5 iterations. The imputation model included age, sex, race and ethnicity, smoking, hypertension, diabetes, PhenoAge acceleration, KDMAge acceleration, survey cycle, and log-transformed fasting weight. CKM stage, education, marital status, Poverty Income Ratio, and alcohol use were imputed using polytomous regression, proportional-odds regression, polytomous regression, predictive mean matching, and logistic regression, respectively. Survey-weighted logistic models were fitted within each imputed dataset using WTSAF2YR/3, SDMVSTRA, SDMVPSU, and survey cycle, with estimates combined according to Rubin's rules. Participant-level imputation was not used to reconstruct biological-age measures for the two entire survey cycles absent from the retained biological-age source.

**References:**

[1] Gao X, Geng T, Jiang M, Huang N, Zheng Y, Belsky DW, Huang T. Accelerated biological aging and risk of depression and anxiety: evidence from 424,299 UK Biobank participants. Nat Commun. 2023 Apr 20;14(1):2277. doi: 10.1038/s41467-023-38013-7. Erratum in: Nat Commun. 2023 Sep 25;14(1):5970. doi: 10.1038/s41467-023-41786-6. PMID: 37080981; PMCID: PMC10119095.

[2] Levine ME, Lu AT, Quach A, Chen BH, Assimes TL, Bandinelli S, Hou L, Baccarelli AA, Stewart JD, Li Y, Whitsel EA, Wilson JG, Reiner AP, Aviv A, Lohman K, Liu Y, Ferrucci L, Horvath S. An epigenetic biomarker of aging for lifespan and healthspan. Aging (Albany NY). 2018 Apr 18;10(4):573-591. doi: 10.18632/aging.101414. PMID: 29676998; PMCID: PMC5940111.

[3] Klemera P, Doubal S. A new approach to the concept and computation of biological age. Mech Ageing Dev. 2006 Mar;127(3):240-8. doi: 10.1016/j.mad.2005.10.004. Epub 2005 Nov 28. PMID: 16318865.

[4] Kwon D, Belsky DW. A toolkit for quantification of biological age from blood chemistry and organ function test data: BioAge. Geroscience. 2021 Dec;43(6):2795-2808. doi: 10.1007/s11357-021-00480-5. Epub 2021 Nov 2. PMID: 34725754; PMCID: PMC8602613.

[5] Mahalanobis, P. C. Mahalanobis distance. Proc. Natl. Inst. Sci. India 49, 234–256 (1936).

[6] Hastings WJ, Shalev I, Belsky DW. Comparability of biological aging measures in the National Health and Nutrition Examination Study, 1999-2002. Psychoneuroendocrinology. 2019 Aug;106:171-178. doi: 10.1016/j.psyneuen.2019.03.012. Epub 2019 Apr 4. PMID: 30999227; PMCID: PMC6599717.

[7] Duong MT, Bingham BA, Aldana PC, Chung ST, Sumner AE. Variation in the Calculation of Allostatic Load Score: 21 Examples from NHANES. J Racial Ethn Health Disparities. 2017 Jun;4(3):455-461. doi: 10.1007/s40615-016-0246-8. Epub 2016 Jun 28. PMID: 27352114; PMCID: PMC5195908.

[8] Wang RH, Wen WX, Jiang ZP, Du ZP, Ma ZH, Lu AL, Li HP, Yuan F, Wu SB, Guo JW, Cai YF, Huang Y, Wang LX, Lu HJ. The clinical value of neutrophil-to-lymphocyte ratio (NLR), systemic immune-inflammation index (SII), platelet-to-lymphocyte ratio (PLR) and systemic inflammation response index (SIRI) for predicting the occurrence and severity of pneumonia in patients with intracerebral hemorrhage. Front Immunol. 2023 Feb 13;14:1115031. doi: 10.3389/fimmu.2023.1115031IF: 5.7 Q1 . PMID: 36860868; PMCID: PMC9969881.
